# Inappropriate administration of mitomycin in a cataract patient

**DOI:** 10.1016/j.ajoc.2025.102357

**Published:** 2025-05-15

**Authors:** Gibran Merchant, Aaron DeWeerd, Jackson Mooney, Manahil Khan, Dasa V. Gangadhar

**Affiliations:** Kansas Health Science University, Kansas College of Osteopathic Medicine, 217 E. Douglas Ave, Wichita, KS, 67202, USA

## Abstract

**Purpose:**

This case report describes the improper administration of intracameral mitomycin after cataract extraction in an 83-year-old male, demonstrating the need for system improvements to prevent “never events.”

**Observations:**

An 83-year-old male presented with visually significant bilateral cataracts, (right eye (OD): 20/80, left eye (OS): 20/70). He underwent technically uncomplicated cataract surgery in the right eye. At postoperative visit one, vision was 20/250 OD, less than anticipated for unclear reasons. On subsequent visits, the patient was found to have worsening photophobia, increasing conjunctival inflammation, corneal edema, and an elevated intraocular pressure of 27 mmHg. Given the variety and complexity of symptoms despite an otherwise uncomplicated surgery, an investigation was launched. An investigation for Toxic Anterior Segment Syndrome (TASS) revealed a mix-up between Cefuroxime and Mitomycin during medication preparation and administration. It was determined that mitomycin was inadvertently injected intracamerally into the patient's eye, causing intraocular toxicity and ultimately, loss of vision.

**Conclusions and importance:**

This case underscores the significance of system failures in the healthcare environment and how “never events” may occur even with appropriate protocols in place. The improper administration of mitomycin emphasizes the need for enhanced safety measures, including improved medication labeling, consistent use of time-outs, and reinforcing their importance in high-volume environments. System changes are essential to reduce the risk of errors and protect patient safety. The use of off-label, compounded medications should be minimized when possible. Ophthalmologists and the pharmaceutical industry should continue to seek an FDA-approved antibiotic for intraocular use in cataract surgery.

## 1 Introduction

This unique case report describes the inadvertent administration of intracameral mitomycin after cataract extraction in an 83-year-old male.

Intracameral antibiotic injections have become a standard step in cataract surgery to minimize the risk of postoperative endophthalmitis. We report a case where a protocol breach led to inappropriate administration of intracameral mitomycin, resulting in permanent vision loss.

Cataract surgery is the most commonly performed surgery in the United States.^1^ The use of intracameral antibiotics to reduce the risk of postoperative infection has become commonplace.^2^ Mitomycin, due to its extreme intraocular toxicity, should never be used during cataract surgery. It is, however, frequently used in other ocular surgeries (glaucoma, pterygium),^3^ necessitating its storage at ambulatory surgery centers (ASC).

### 1.1 Report of a case

An 83-year-old male presented for an eye exam with visually significant bilateral cataracts where right eye vision (OD) was 20/80 and left eye vision (OS) was 20/70. Following technically uncomplicated cataract surgery in the right eye using a monofocal aspherical implant (Alcon Surgical, SN60WF), the patient presented on postoperative day #1 with vision of 20/250 OD, moderate intraocular inflammation, and corneal edema.

**Fig. 1:**
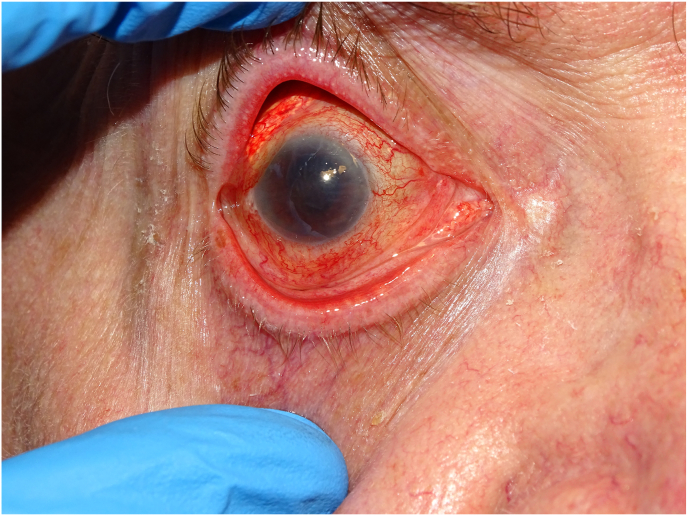

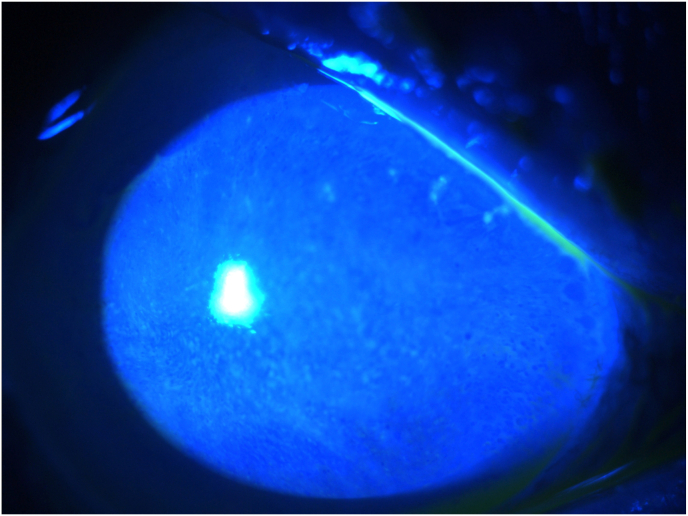

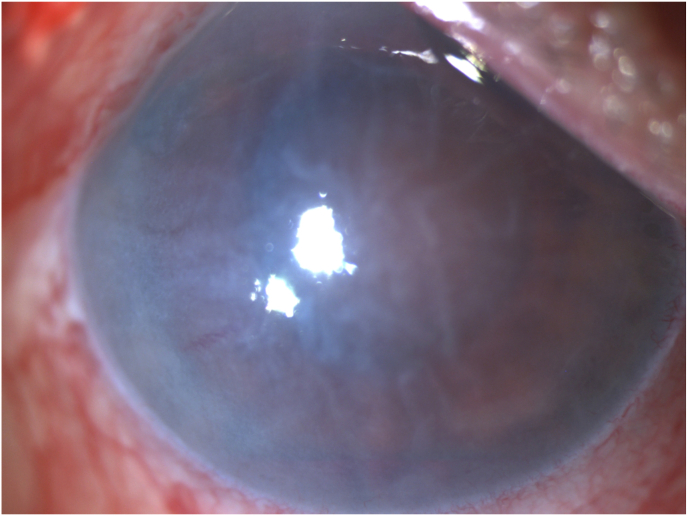
One month post cataract surgery, the patient continues to manifest significant inflammation (A) and has developed severe corneal edema (B,C).

By day 4, the patient developed significant photophobia, increased conjunctival inflammation, worsening corneal edema, and elevated intraocular pressure to 27 mmHg. The surgeon noted two 2mm corneal abrasions and applied a therapeutic soft lens. On day 5, the soft lens was removed, and worsening corneal edema and intraocular inflammation were observed. Due to concerns for endophthalmitis, a retinal consult was sought. On day 7, the patient was treated with intravitreal antibiotics and steroids per infection protocol. Vitreous cultures remained negative. Due to an atypical presentation and clinical course with a negative infectious workup, the ASC launched an investigation for causes of possible toxic anterior segment syndrome (TASS) (see Fig. 1).

At nine days post-cataract surgery, the completed investigation found an extra vial of antibiotic and a shortage of one vial of mitomycin. These findings, combined with thorough interviews of all involved staff, determined that 0.1 cc's of mitomycin (0.4mg/mL) had most likely been inadvertently delivered and injected into the patient's eye. The patient was informed and provided details of the investigation. Vision OD continued to deteriorate to hand motions due to worsening corneal edema and intraocular inflammation. The medical team deemed surgical intervention unfeasible, opting for aggressive topical steroids and monitoring via serial ultrasounds.

At the one-month follow-up, 3+ corneal edema persisted. The patient developed a choroidal detachment which was noted on B-scan by a vitreoretinal-trained surgeon. The treating physicians decided against further surgical intervention at this time. No intervention was undertaken until the six-month mark, when the inflammation had resolved, and the retina remained attached on ultrasound. The patient underwent a corneal transplant (DSEK) with clearing of the edema. Despite a clear cornea, the patient's vision remained at the hand motions level. Within several months, the DSEK failed, and the corneal edema recurred. Repeat ultrasound revealed retinal detachment. Due to chronic hypotony and limited prognosis, the treating physicians opted for no further intervention as it was felt that mitomycin toxicity had led to irreversible ocular damage.

## 2 Discussion

Mitomycin is a potent alkylating agent that cross-links DNA, inhibiting DNA replication and thereby inducing apoptosis. Ocular tissue is extremely sensitive to this toxin. Intravitreal injection of mitomycin in animal studies has shown Müller cell degeneration followed by death of photoreceptors and retinal pigment epithelium cells.^4^ In a reported case of inadvertent intravitreal mitomycin injection, extensive toxicity was evidenced by diffuse retinal ischemia as seen on fluorescein angiography demonstrating minimal or no retinal/choroidal perfusion.^5^

**Fig. 2:**
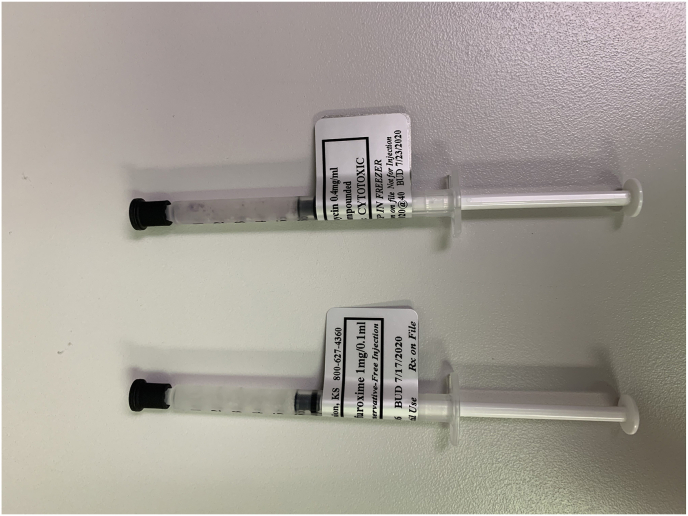

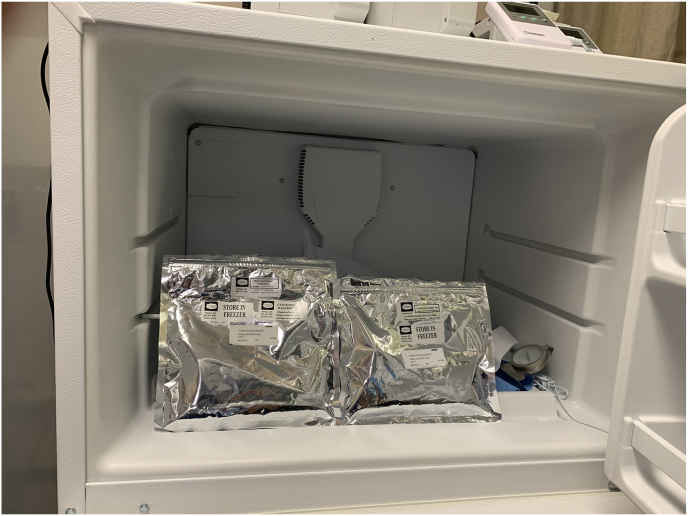

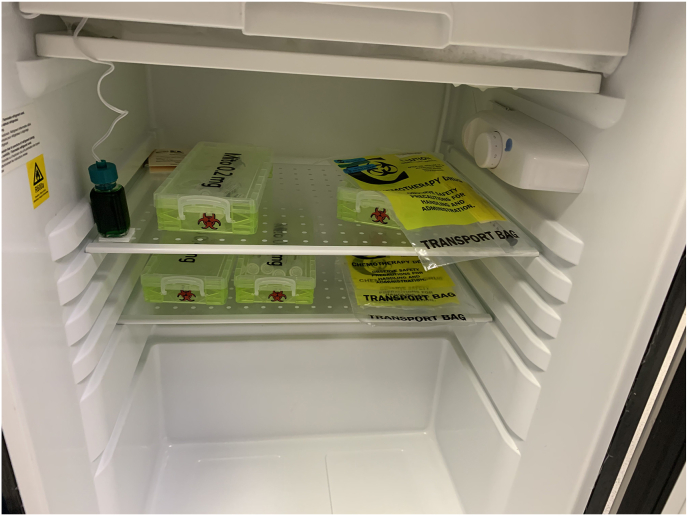
Cefuroxime (left) and Mitomycin (right) with their respective labels at the time of the incident. Note the minimal identifying characteristics of the syringes (A). Packets of both Cefuroxime (left) and Mitomycin (right) in the same refrigerator at the ASC. A recent switch to a new compounding pharmacy resulted in the medications having similar packaging (B). After an ASC system redesign, the new location of an additional refrigerator with the implementation of high-visibility yellow boxes where the Mitomycin is now stored within the ASC (C).

In this case of mitomycin-induced ocular toxicity, the surgery center kept both chemotherapy drugs and compounded antibiotics in the same refrigerator (Fig. 2A and B). Despite the correct labeling and strict time-out protocols, the circulating nurse inadvertently retrieved the medication from the wrong packet and passed it to a second nurse. Neither nurse noted the improper medication and handed it to the circulating OR technician, who withdrew it into a syringe labeled “Cefuroxime.” When the surgeon received the medication on the Mayo stand, it was labeled Cefuroxime making an appropriate distinction impossible for the operating surgeon.

In this case, a system failure led to the misadministration of mitomycin. This case illustrates that despite there being systems in place at the ASC such as the WHO checklist, “time out” protocols, and an experienced surgeon with over 40,000 cataract surgeries in 35 years, such “never events” can still happen. This can be due to factors such as complacency, boredom, and redundancy which can easily occur in a high-volume surgical environment. The potential for human error can never be fully eliminated. Hence, systems must be in place to minimize the opportunity for human error.

The ASC has since implemented changes, including relocating chemotherapeutic agents to a different part of the building and adding extensive colored labeling to the chemotherapeutic agents (Fig. 2C). Additionally, routine reminders and training in medication verification and diligent use of “time outs” were incorporated into the operations of the surgery center.

This case also illustrates the risks posed by compounded medications having similar labeling. Although the use of intracameral antibiotics at the conclusion of cataract surgery has gained widespread acceptance, the lack of an FDA-approved intracameral antibiotic in the U.S. continues to frustrate surgeons.

“Never events” continue to occur in medicine despite our decades-long recognition of such failures.^6^ In 1999, the IOM published a report where they stated that “the problem is not bad people in health care – it is that good people are working in bad systems that need to be made safer.“^7^ Therefore, instead of assigning individual blame, system changes with thoughtful redundancies are necessary to reduce medical errors and minimize the risk of patient harm.^7^

## CRediT authorship contribution statement

**Gibran Merchant:** Writing – review & editing, Writing – original draft, Visualization. **Aaron DeWeerd:** Writing – review & editing, Writing – original draft. **Jackson Mooney:** Writing – original draft. **Manahil Khan:** Writing – original draft. **Dasa V. Gangadhar:** Writing – review & editing, Writing – original draft, Supervision.

## Patient consent

Written consent to publish this case has not been obtained. This report does not contain any personal identifying information.

## Authorship

All authors attest that they meet the current ICMJE criteria for Authorship.

## Funding

No funding or grant support

## Declaration of competing interest

The authors declare that they have no known competing financial interests or personal relationships that could have appeared to influence the work reported in this paper.
